# Colorectal Cancer and Basement Membranes: Clinicopathological Correlations

**DOI:** 10.1155/2014/580159

**Published:** 2014-12-28

**Authors:** Charalampos C. Mylonas, Andreas C. Lazaris

**Affiliations:** 1st Department of Pathology, School of Medicine, National and Kapodistrian University of Athens, 11527 Athens, Greece

## Abstract

Colorectal cancer (CRC) is the third most commonly diagnosed cancer in males and the second in females. In 2008, an estimated 1.2 million people were diagnosed with and 608,700 people died of CRC. Besides diagnosis and treatment, prognosis is an important matter for cancer patients. Today, clinicopathological correlations have many applications in cancer prognostication. Examples include the prediction of the medium patient survival and the screening for patients suitable for specific therapeutic approaches. Apart from traditional prognostic factors, such as tumor stage and grade, new markers may be useful in clinical practice. Possible markers may result from the study of basement membranes (BMs). BM seems to play a role in the pathogenesis of colorectal cancer, so BM alterations may have prognostic significance as well. The purpose of this review is to briefly describe BMs and their relationship with CRC, in the aspect of clinicopathological correlations.

## 1. Introduction

Colorectal cancer (CRC) is a malignant neoplasm, situated in the colon or rectum. As proposed by Fearon and Vogelstein [1], CRC results from acquired and/or hereditary genetic alterations of the colonic mucosa. In this model, transition from normal epithelium to benign adenomatous lesions and eventually adenocarcinoma is induced by the accumulation of critical mutations. Epigenetic alterations also seem to contribute to the process [2].

CRC poses a major public health problem, being the third most commonly diagnosed cancer in males and the second in females. In 2008, over 1.2 million people were diagnosed with and 608,700 people died of CRC [3]. As with all forms of cancer, it is now considered to be a chronic systematic disease. At first, the neoplastic cells develop at the primary tumor site and then metastasize (via lymphatic or blood vessels) to other sites of the body [4, 5].

With respect to CRC, clinical decision making is mainly driven by tumor staging, as reflected by the AJCC/UICC TNM-classification. Until today, it remains the gold standard for tumor evaluation and risk assessment. It is based on several histopathological and clinical criteria, including local tumor extent, regional node involvement, and distal metastasis [6]. Additional prognostic markers are tumor border configuration, tumor budding, and tumor grade [6]. Another classification system is the Dukes staging system. Widely used in the past, it is less detailed but, as with AJCC/UICC TNM-classification, it takes into consideration the local invasion of the tumor, lymph node involvement, and distal metastasis [7, 8]. However, even with the use of the TNM-classification method, there are some patients of lower TNM-stages that have a worse clinical outcome than patients of higher TNM-stages [9].

In fact, CRC is a whole group of diseases, rather than a single one. CRC tumors differ not only in the grade of differentiation and cancerous potential, but also in the genetic mutations involved and the expression of molecular markers [10–12]. Thus, the need for an accurate diagnosis, prognosis, and efficient therapeutic approach has led scientists to search further into the molecular level. This may reveal new prognostic markers, thus aiding the existing tumor classification systems in determining CRC prognostication.

From a histological point of view, one can see that tumors consist of more than just neoplastic cells. Besides the latter, there is also the tumor stroma: fibroblasts, immune cells, blood and lymphatic vessels, and more, all surrounded by what is called the extracellular matrix (ECM) [13]. Interaction between the tumor stroma and the neoplastic cells regulates all aspects of tumorigenicity [13–15].

The specialized structure of the ECM which separates parenchymal cells from stromal tissues is called the basement membrane (BM). The latter consists of thin extracellular matrices, mostly composed of proteins, glycoproteins, and glycosaminoglycans [16–19]. The neoplastic cells, in order to metastasize, must not only break through their own BM (in epithelial-derived tumors) but also the BM of lymphatic and blood vessels [4, 5]. Further understanding of this complex phenomenon not only is of scientific interest, but may result in useful clinical applications.

The purpose of this review is to briefly describe BMs and their relationship with CRC, in the aspect of clinicopathological correlations.

## 2. What Is the Basement Membrane?

As mentioned above, the BMs are thin extracellular matrices, mostly composed of proteins, glycoproteins, and glycosaminoglycans. More specifically, they are primarily composed of type IV collagen, laminin, entactin/nidogen, and perlecan (heparan sulfate proteoglycan) [17–19]. However, they also contain a variety of growth factors and other molecules [20]. With the use of the electron microscope in glutaraldehyde-fixed and heavy-metal-impregnated thin sections, the BM can be divided into two distinct parts [17]: (1) lamina lucida, immediately adjacent to the parenchymal cells, and (2) lamina densa, right beneath lamina lucida. This morphology has been questioned by studies using rapid freeze-substitution technique, in which the BMs appear solely as lamina densa [21, 22].

It must be noted that although the basic ultrastructure of the BM is relatively identical in all tissues, differences do exist. This heterogeneity derives partly from the several trimer combinations of the respective laminin and type IV collagen chains. At least 16 laminin [23] and 6 collagen IV [24, 25] isoforms are currently known. In addition, tissue-specific differences in minor proteins and carbohydrate components also contribute to the heterogeneity [17]. These differences may account for the different roles of the BMs in each tissue.

BMs contact epithelial and endothelial cells, fat, smooth muscle and Schwann cells, and more, appearing to act in many ways [17]. Firstly, they provide an intermediate adhesion area between parenchymal cells and the interstitial matrix. Moreover, they act as a molecular filter, regulating the passage of substances within it, mostly due to the glycosaminoglycans. Also, they hold an important role in cellular organization and differentiation, through mutual interaction between cell surface receptors and adjacent ECM components.

## 3. How Is Basement Membrane Involved in Cancer?

Given the normal function of the BMs, it would be interesting to look into the changes which occur during cancer. As aforementioned, in order for the malignant cells to metastasize, they must not only break through their own BM (in epithelial-derived tumors), but also the BM of lymphatic and blood vessels [4, 5]. In order to do so, they must first attach to the BM components. This is done via integrins and other cellular attachment molecules [5, 26, 27]. This is followed by the lysis of the BM components by tumor- or stroma-cell derived proteases, like matrix metalloproteinase-9 [28–30], the tumor cells consequently moving through space created in the BMs. A point of interest is the fact that the BM acts more than a mere barrier for the malignant cells. Many degradation products of BM components seem to have angiogenic, angioinhibitory, growth, and chemotactic properties [5, 31–33]. Growth factors embedded within the BM are released upon its degradation, such as VEGF [13, 34]. All these may drastically affect the tumor behaviour, regulating angiogenesis, tumor growth, and migration through the interstitial matrix.

One component of the BMs rigorously studied for its relationship with CRC is laminin-332 gamma-2 chain (LN*γ*2). This molecule is part of the laminin-332 isoform, a heterotrimer comprised of *α*3, *β*3, and *γ*2 chains [35–38]. Laminin-332 isoform is found in the BM of normal intestinal mucosa [39–41]. It is one of the few laminin isoforms which contain the *γ*2 chain [23, 35–38, 42]. Studies have shown that whereas colonic adenomas maintain their continuous laminin-332 expression, the transition to a carcinoma and metastatic lesion is associated with discontinuous expression of laminin-332 and even an abnormal expression of the *β*3 and *γ*2 subunits in the cytoplasm of a subset of carcinoma cells [41, 43, 44]. It has been proposed that, during carcinogenesis, deregulation of the Wnt signaling pathway [45] leads to an increase of the cytoplasmic expression of the LN*γ*2 at the invasive front of the tumor [11, 46]. The latter lessens laminin-332 expression in the BM and thus destabilizes cell-matrix adhesion, leading to its detachment from the BM and facilitating migration [47, 48]. Moreover, the excess LN*γ*2 may be cleaved by matrix metalloproteinases into degradation products, which promote cell migration and/or invasion [49–51]. As proposed, these fragments act through the activation of the epidermal growth factor receptor pathway [52].

In addition to this, BMs seem to play a role in the epithelial-to-mesenchymal transition, a process which is thought to be involved in the metastatic process of CRC [53, 54]. This process seems to take place where the tumor contacts invaded tissue, thus the invasive front. There, a dedifferentiation of neoplastic epithelial tumor cells towards a mesenchymal-like phenotype seems to take place, with less expression of BM components and intercellular adhesion molecules. BM components and epithelial phenotype seem to be maintained in the central tumor mass. To the contrary, metastases from such tumors seem to undergo the opposite process, thus mesenchymal-to-epithelial retransition [55]. The latter involves redifferentiation and expression of BM components, which is proposed to facilitate metastatic growth, since mesenchymal-like phenotype seems to be linked to a growth arrest in CRCs [56].

## 4. Why Study the BMs?

So, we come to the question: Why should someone be interested in BMs and their relationship to CRC? Well, in the aspect of clinicopathological correlations, the aim is to use pathological findings to aid clinical practice. Thus, ways to aid diagnosis, prognosis, therapy, and even prevention of the disease are being sought. Considering the role that BMs apparently play in cancer, they may prove fruitful for the above purposes. For example, since the tumors must penetrate the BM before metastasizing [4, 5, 26, 27], the study of BM components at the invasive front may pertain to tumor malignancy, thus obtaining information about survival and metastatic potential. Moreover, since components of the BM seem to affect tumor growth and angiogenesis [13, 32, 34], the expression pattern of these components may be associated with tumor sensitivity to therapeutic approaches and implemented as a screening method, in order to find which patients are suitable for a specific therapy. These examples indicate that there are hypotheses worth investigating.

## 5. Clinicopathological Correlations

There are many studies in bibliography which examine the relationship between BMs and CRC, in the aspect of clinicopathological correlations. The main methodology requires paraffin sections of primary CRCs or metastases from lymph nodes and other organs [57]. The samples are immunohistochemically stained using antibodies for specific BM components. Then, clinical outcomes like “survival status” or “response to therapy” are compared between patients expressing specific BM components and patients lacking that expression, the aim being to find statistically significant correlations between the variables “biomarker expression” and “clinical outcome,” while avoiding the spurious correlations. The latter are caused by confounding factors, such as the tumor's stage, which (as reflected by the AJCC/UICC TNM-system) is the gold standard for tumor evaluation and risk assessment [6]. Of course, the appreciation of a correlation in everyday practice must be confirmed by multiple studies for it to eventually become a standardized procedure. Moreover, technical issues may need to be addressed. For example, as stated in several papers, one knows that several antibodies do not work or work poorly on formalin-fixed material [58], and this may strengthen the difficulty in having a standardized procedure to assess BM component expression in clinics.

Some of the most common markers studied are (1) loss of BM integrity and (2) expression of LN*γ*2, both at the invasive front of the primary tumor. Besides these two, several others have been studied, some of which are mentioned in this review, which focuses on biomarkers related to laminins and collagen IV. It is worth mentioning, however, that many other factors related to the BMs have also been examined as potential markers for CRC progression, such as matrix metalloproteinase-2 [59], matrix metalloproteinase-9 [60], and urokinase plasminogen activator receptor [61], all of which are involved in the degradation of the BM.

### 5.1. Loss of BM Integrity at the Invasive Front

Taking into account all the aforementioned, it is reasonable to assume that tumors with a greater loss of BM integrity at the invasive front may have a worse prognosis compared to those with a relative continuity.

Indeed, loss of BM integrity at the invasive front of primary CRC tumors is likely to have prognostic significance. Total loss or considerable discontinuity of the BM has been associated with a higher metastatic potential [58, 62], poor survival status [57, 58], and less differentiated tumors [57, 62]. Abnormal accumulation of laminin in the cytoplasm of tumor cells at the invasive front seems to have prognostic significance as well [62].

More specifically, Lazaris et al. [57] examined a series of 151 CRC cases, assessing the immunohistochemical expression of laminin and collagen IV at the invasive front. They found that discontinuity in their BM expression was associated with less differentiated tumors and a worse 3-year survival status. The relationships reached statistical significance. However, no association between BM continuity and the stage of disease was noticed.

The results are in concordance with Delektorskaya and Kushlinskii [62], who studied 264 biopsy specimens from primary CRC. They too examined the invasive front of the tumors for laminin and collagen IV, finding that abnormal accumulation of laminin in the cytoplasm of tumor cells is correlated with higher local invasion according to Dukes staging and higher metastatic potential. Moreover, loss of collagen IV-containing BMs was more frequently observed in metastasizing and low-differentiated tumors.

Spaderna et al. [58] used a collection of 125 cases of pT3M0 R0 CRCs to examine BM expression at the invasive front and laminin *α*3 chain as an indicator for BM integrity. Selective loss of *α*3 chain-containing BMs at the invasive front strongly correlated with distant metastasis and a worse 5-year survival rate. However, in the multivariate Cox regression analysis, BM loss did not reach statistically significant levels as an independent prognostic factor.

The above studies indicate the possible significance of BM integrity loss at the invasive front of primary CRC tumors as a possible prognostic marker for metastatic potential and patient survival. Of course, more studies need to be carried out with larger numbers of patients, in order to verify and support these results. Also, a way to standardize the measurement of BM integrity must be used, and since laminin and collagen IV have many isoforms [23–25], those measured in each study should be specified.

### 5.2. Expression of Laminin-332 Gamma-2 Chain at the Invasive Front

According to the aforementioned, LN*γ*2 expression at the invasive front seems to play an important role in CRC cell migration and/or invasion [49–51]. Thus, it may prove itself as a useful prognostic marker for metastatic potential.

Indeed, LN*γ*2 expression at the invasive front seems to greatly influence the degree of clinical aggressiveness of CRC and its tendency to metastasize [63–65]. High expression of LN*γ*2 has been strongly correlated with synchronous liver metastasis [63] and a worse survival status [63–65].

More specifically, Aoki et al. [63] examined a series of 103 stages II, III, and IV CRC cases, assessing the immunohistochemical expression of LN*γ*2 by tumor cells and finding that expression was higher at the invasive front of the tumors and that this was significantly associated with synchronous liver metastasis and a worse survival rate. The latter was confirmed by univariate and multivariate analysis.

Lenander et al. [64] examined 93 CRC cases (Dukes stages A–C) for LN*γ*2 expression. Univariate analysis identified LN*γ*2, tumor differentiation, and Dukes stage as significant variables in predicting prognosis. However, by multivariate analyses, this study could not demonstrate that LN*γ*2 expression was an independent predictive factor for survival. Only Dukes stage was identified as a significant covariate.

Shinto et al. [65] examined a series of 120 pT3 primary CRC cases. In order to assess the expression of LN*γ*2 they used tissue microarrays, a technique which allows for multiplex histological analysis. What they found was that LN*γ*2 expression had prognostic significance solely at the invasive front of the tumor and not at other sites, such as the central mass, and was strongly associated with the 5-year survival of the patients. More specifically, patients with a high LN*γ*2 expression at the invasive front had a 5-year survival rate of approximately 55%, while patients with low expression had a 5-year survival rate of approximately 80%. This difference was statistically significant, and multivariate analysis identified LN*γ*2 expression as an independent prognostic factor in addition to nodal and distal metastasis.

The studies mentioned above support the possible prognostic significance of LN*γ*2 expression at the invasive front of CRC tumors. Of course, more studies are required in order to determine whether this is an independent prognostic factor or related to others, such as tumor stage and differentiation. A standard protocol with specific probes may prove helpful in minimizing inhomogeneities between studies.

### 5.3. Other Markers

Besides loss of BM integrity and LN*γ*2 expression at the invasive front, many other markers have been examined for their prognostic significance in CRC, with studies supporting them being fewer however. Such examples are mentioned below.

- As noted above, LN*γ*2 may promote cell migration and/or invasion [49–51]. Lenander et al. [66] examined this molecule as an indicator of incipient malignant transformation of benign colorectal adenomas, using 67 cases of nonmalignant polyps of different types and assessing them for LN*γ*2 expression. The results showed that LN*γ*2 expression became progressively more frequent from hyperplastic polyps (0% expressed *γ*2 chain) to tubular (12,5%), serrated (17%), and villous (25%) adenomas.
- LN*γ*2 has also been investigated as a marker to identify ulcerative colitis patients with increased risk of cancer development. Habermann et al. [67] studied 8 patients with ulcerative colitis-associated cancers in comparison with 16 cancer-free patients with other risk factors for CRC, such as duration of the disease. By retrospective evaluation, they found a higher frequency of LN*γ*2 expression in the first group, and the difference reached statistical significance.
- Other researchers have focused on the prognostic significance of BM components in tissues other than the primary tumor. Ogawa et al. [68] studied laminin and collagen IV expression in the lymph channel and in vascular vessels. They found that patients with synchronous or metachronous metastasis expressed laminin in the lymph channel more frequently than patients disease-free for 5 years. Collagen IV did not differ significantly between these groups. Gulubova and Vlaykova [69] studied 55 cases with synchronous metastasis, in an effort to determine whether metastases with a fibrotic capsule had a longer survival rate than those lacking one. It was shown that the absence of a fibrotic capsule is significantly associated with a worse postsurgery survival. Also, noncapsulated metastases were more often positive for the BM components laminin and collagen IV and other molecules. The expression of these components in the adjacent liver sinusoids was associated with a worse prognosis.
- Jayne et al. [70] assessed the effects of ECM protein expression and response to chemoradiotherapy. The study involved ECM proteins other than those expressed in BMs. The proteins studied were fibronectin and fibronectin receptor (*α*5*β*1 integrin). However, the researchers used laminin and collagen IV upon comparison and analysis of 32 pretreatment rectal cancer biopsies. It seemed that neither laminin nor collagen IV expression was correlated with response to chemoradiotherapy.
- Besides laminin-332, other laminins have also been implicated in CRC. These include laminin-111 [71] and laminin-511 [72]. Studies supporting their role in CRC, however, are mostly limited to in vitro and animal studies. More specifically, as shown by studies in mice, laminin-111 seems to enhance the malignant phenotype of CRC cells, probably acting as a chemoattractant of stromal and vascular cells [73]. Laminin-511 has been shown to contribute to the motility of colon cancer cell line LIM1215, in cooperation with epidermal growth factor receptor [74]. If these laminins are proven to play a determinant role in CRC, then it is reasonable to study their prognostic significance.

The previous studies underline the need for further investigation of BM components as prognostic markers. BM components of the lymph channel [68] and distal metastases [69] appear to have a prognostic significance as well. Also, BM component expression may play a role in screening of premalignant conditions, such as colorectal adenomas [66] and ulcerative colitis [67]. Altogether, studies aiming at BM components will shed light on whether the expression of such molecules is related to response to specific therapeutic approaches. Focusing on other laminins besides laminin-332 may also result in useful prognostic markers.

## 6. Conclusions

Alterations in BMs seem to be implicated in the progression of CRC, and some of these alterations can be identified by immunohistochemical evaluation. This renders them possible prognostic markers. Moreover, many studies suggest that BM components expression may indeed play a prognostic role in CRC. Determining the metastatic potential, survival status, response to therapy, and screening of benign lesions are a few of the possible clinical applications. Before reaching this point, however, more research is needed. Future studies should be focused on (1) quantifying the BM component expression and (2) reducing inhomogeneities between research methods. This will probably reduce the discrepancies between the results and clarify whether or not these markers should enter everyday clinical practice.

## Abbreviations

CRC: Colorectal cancer
BM: Basement membrane
ECM: Extracellular matrix
LN*γ*2: Laminin-332 gamma-2 chain.
